# Comorbidity of migraine with ADHD in adults

**DOI:** 10.1186/s12883-018-1149-6

**Published:** 2018-10-16

**Authors:** Thomas Folkmann Hansen, Louise K. Hoeffding, Lisette Kogelman, Thilde Marie Haspang, Henrik Ullum, Erik Sørensen, Christian Erikstrup, Ole Birger Pedersen, Kaspar René Nielsen, Henrik Hjalgrim, Helene M. Paarup, Thomas Werge, Kristoffer Burgdorf

**Affiliations:** 1 Danish Headache Center, Department of Neurology, Rigshospitalet Glostrup, University Hospital of Copenhagen, Copenhagen, Denmark; 2 Novo Nordisk Foundation Center for Protein Research, Faculty of Health and Medical Sciences, University of Copenhagen, Copenhagen, Denmark; 3 Department of Clinical Immunology, the Blood Bank, Rigshospitalet, University Hospital of Copenhagen, Copenhagen, Denmark; 4 Department of Clinical Immunology, Aarhus University Hospital, Aarhus, Denmark; 5 Department of Clinical Immunology, Naestved Hospital, Naestved, Denmark; 6 Department of Clinical Immunology, Aalborg University Hospital, Aalborg, Denmark; 7 Department of Epidemiology Research, Statens Serum Institut, Copenhagen, Denmark; 8 Department of Hematology, Copenhagen University Hospital, Rigshospitalet, Copenhagen, Denmark; 9 Department of Clinical Immunology, Odense University Hospital, Odense, Denmark; 10 Institute of Biological Psychiatry, Mental Health Centre Sct. Hans, Copenhagen University Hospital, Roskilde, Denmark; 11 Department of Clinical Medicine, University of Copenhagen, Copenhagen, Denmark; 12 The Lundbeck Foundation Initiative for Integrative Psychiatric Research, iPSYCH, Copenhagen, Denmark; 13 Danish Headache Center, Neurological department, Copenhagen University Hospital, Nordre Ringevej 69, DK-2600 Glostrup, Denmark

**Keywords:** Migraine, Attention deficiency and hyperactivity disorder, Comorbidity

## Abstract

**Background:**

Migraine and Attention Deficit and Hyperactivity Disorder (ADHD) have been found to be associated in child and adolescent cohorts; however, the association has not been assessed in adults or otherwise healthy population. Assessing the comorbidity between ADHD and migraine may clarify the etiopathology of both diseases. Thus, the objective is to assess whether migraine (with and without visual disturbances) and ADHD are comorbid disorders.

**Methods:**

Participants from the Danish Blood Donor Study (*N* = 26,456, age 18–65, 46% female) were assessed for migraine and ADHD using the ASRS ver 1.1 clinically validated questionnaire and self-reported migraine in a cross-sectional study. Logistic regression was used to examine the comorbidity between migraine and ADHD, and their associated endophenotypes.

**Results:**

Migraine was strongly associated with ADHD (OR = 1.8, 95% CI = 1.5–2.1), (238/6152 vs 690/19,376). There was a significant interaction between age and gender, with comorbidity increasing with age and female sex. Post-hoc analysis showed that migraine with visual disturbance was generally associated with a marginally higher risk of ADHD and this was independent of ADHD endophenotypes.

**Conclusion:**

Migraine and ADHD were demonstrated to be comorbid disorders; the association with ADHD was most prominent for participants with migraine with visual disturbances. Future studies will elucidate which genetic and environmental factors contribute to migraine-ADHD comorbidity.

## Background

Migraine is a complex and multifactorial headache disorder with a lifetime prevalence of 16–18% [1–3]. Migraine is twice as prevalent in females, and onset is typically between adolescence and the late 50s [1–3]. According to the World Health Organization (WHO), migraine is the sixth most disabling disease in the world with high financial costs to society [4]. Response to acute treatment varies considerably and approximately 20% of the pharmacologically treated patients experience no symptom relief after medication [5]. There are two major endophenotypes in migraine: migraine with or without aura. Aura is a sensory disturbance, which is predominantly seen as visual disturbances (99%) with a subsequent headache or migraine [6, 7].

Attention deficit and hyperactivity disorder (ADHD) is characterized by inappropriate levels of inattention, e.g., difficulty keeping attention, keeping track of details, and difficulty structuring trivial duties or following instructions, hyperactivity, e.g., speaks a lot, difficulty relaxing or sitting still, and impulsivity, e.g., often interrupts other people during conversations or answers a question before the question is finished [8]. In contrast to migraine, ADHD has an early onset and a pooled worldwide prevalence of 5.3% in child and adolescent populations [9]. The current treatment strategies do not completely remove the symptoms in both children and adults and approximately 30% of all patients do not respond to medical treatment or develop serious adverse reactions [10, 11].

It is well established that migraine is comorbid with psychiatric traits, in particular depressive and bipolar disorder [12, 13], and that the comorbidity is partly explained by shared genetics [14]. More recently, the comorbidity between migraine and ADHD has also been assessed [15–18]. In adults, a clinical case-control study of ADHD (*n* = 572/675) found an increased prevalence of migraine when compared to community controls [16], and subsequently the same investigators showed a positive association between prescription of anti-migraine and anti-ADHD drugs to adults in the total Norwegian population(*n* > 4mill) [17]. In line with this, Arruda et al. reported a higher prevalence of ADHD among children with migraine (5–12 years) than for non-headache individuals in a pediatric population cohort (*n* = 5671) [19]. According to the recently published meta-analysis including child and adolescence studies and the Fasmer et al. adult study, there is a positive association between migraine and ADHD with odds-ratio of 1.3 [16, 20].

Using a cross-sectional study of adults (age 18–65 years), we test the hypothesis that migraine is comorbid with ADHD in 26,456 participants using clinically validated questionnaires.

## Methods

### Participants

From November 2015 to September 2017 voluntary blood donors were recruited as part of the Danish Blood Donor Study (DBDS) (www.DBDS.dk). In brief, the DBDS was initiated in 2010 and is an ongoing prospective research cohort and biobank that recruits participants between 17 and 67 years of age from blood banks across Denmark [21, 22]. Individuals in chronic medical treatment or frequent travelers to countries considered resulting in high-risk of blood disease are not allowed to participate. Approximately 95% of all invited individuals are consented to participate in DBDS [22]. At enrolment, each individual gives oral and written informed consent to participate in DBDS and subsequently answers a digital tablet-based questionnaire including a migraine (two questions) and an ADHD (18 questions) module. In total, 29,489 participants were recruited to the study. The study is approved by the Danish Data Protection Agency (2007–58-0015) and the Ethical Committee of Central Denmark (M-20090237). For further details about the DBDS platform and questionnaire, see Pedersen et al. [22] or Burgdorf et al. [21]

In total, 29,489 participants were given a questionnaire. We excluded 3033 individuals due to missing answers on either the SQM (*n* = 2057) or the ASRS items (*n* = 1108) of which *n* = 132 were missing both. The excluded individuals did not differ significantly with respect to age (chi-square test, *P*-value = 0.24) compared to the study population. The excluded individuals were slightly older (median age: 44 years, IQR = 31–54 years) when compared to the study population (median age: 42 years, IQR = 30–52 years) (Wilcoxon test, *P*-value = 3.7e-8). Importantly, the frequency of migraine in individuals excluded because of missing information in the ASRS questionnaire was similar to that of the study population (24.1%). Further, the frequency of ADHD in individuals excluded because of missing items in the SQM questionnaire was similar to that of the study population 4.2%.

### The migraine module

The presence of migraine was evaluated by two questions from the population screening questionnaire for migraine (SQM). The participants were asked two questions (“Have you ever had migraine?” and “Have you ever had visual disturbances lasting 5-60 min followed by headache?”) of the original SQM. Participants who had a positive response to either of the two questions were considered to have migraine and are referred as migraine cases in this study. Individuals with missing information or who answered “I don’t know” were excluded. A detailed description of the SQM questionnaire can be found elsewhere [23]. In short, the SQM has previously been shown to identify 93% of those with migraine with aura and 75% of those with migraine without aura in a Danish setting [23]. Using the national prescription register, we found that 89.5% of those prescribed migraine-specific treatment, i.e. triptans ATC-codes N02CC01–7, are captured using these two questions.

### The ADHD module

Current self-reported ADHD symptoms were assessed using the current national recommendation for clinical assessment of adults by using the ADHD Self-Report Scale v1.1 (ASRS) [24, 25] translated to Danish Dalsgaard et al. [26]. The ASRS consists of 18 items based on the *Diagnostic and Statistical Manual of Mental Disorders fourth edition* (DSM-IV) diagnostic criteria for ADHD and is the recommended version for clinical use in Denmark. Each of the 18 questions was scored on a five-point Likert scale ranging from never = 0 to very often = 4. In this study we use the optimal Kessler et al. 18-item ASRS scale score obtained by summarizing all items (total range of 0–72) as the primary outcome. The ASRS has been validated in multiple countries using different populations [27–30]. The scale dichotomizes ADHD based upon all items for ADHD (having a score > 36), inattentive subtype (items 1–4 + 7–11, having a score > 23), the hyperactivity-impulsivity subtype (items 5–6 + 12–18, having a score > 23), and the 6-item screening score (having a score > 13) [28]. A more detailed description of the ASRS questionnaire can be found elsewhere [24].

### Statistical analysis

The study population was described by numbers and percentages for categorical variables and as median and interquartile range (IQR) for continuous variables. Differences in distributions between participants with and without migraine and ADHD symptoms were analyzed using Fisher’s exact test or Mann-Whitney/Wilcoxon test (age does not follow a normal distribution, thus non-parametric analysis is used).

Logistic regression was used to analyze the association between migraine and ADHD (dichotomous variables) in the study population, adjusting for age, sex, and the interaction between sex and age, with migraine as the dependent variable. We used a logistic regression model with a binary outcome of either migraine, migraine with visual disturbance, or migraine without visual disturbances. We did not include sampling weights, as these are not available for DBDS. We calculatedthe regression in two ways including age as a quantitative trait or as a categorical trait (10 year interval). We report the derived odds ratios (OR) and 95% confidence intervals (CIs). A *P*-value< 0.05 was considered statistically significant. Post-hoc analyses were performed using different thresholds of the ASRS score (from 0 and 52), and single ASRS items (data not shown). To check if the specified model is appropriate for the data, the predicted and observed residuals were inspected for each analysis and no issues were observed.

All statistical analyses were performed using the statistical package for R version 3.3.3: stat, basic, ggplot2, eeptools, cowplot, and GirdExtra.

## Results

### Study population

**Fig. 1:**
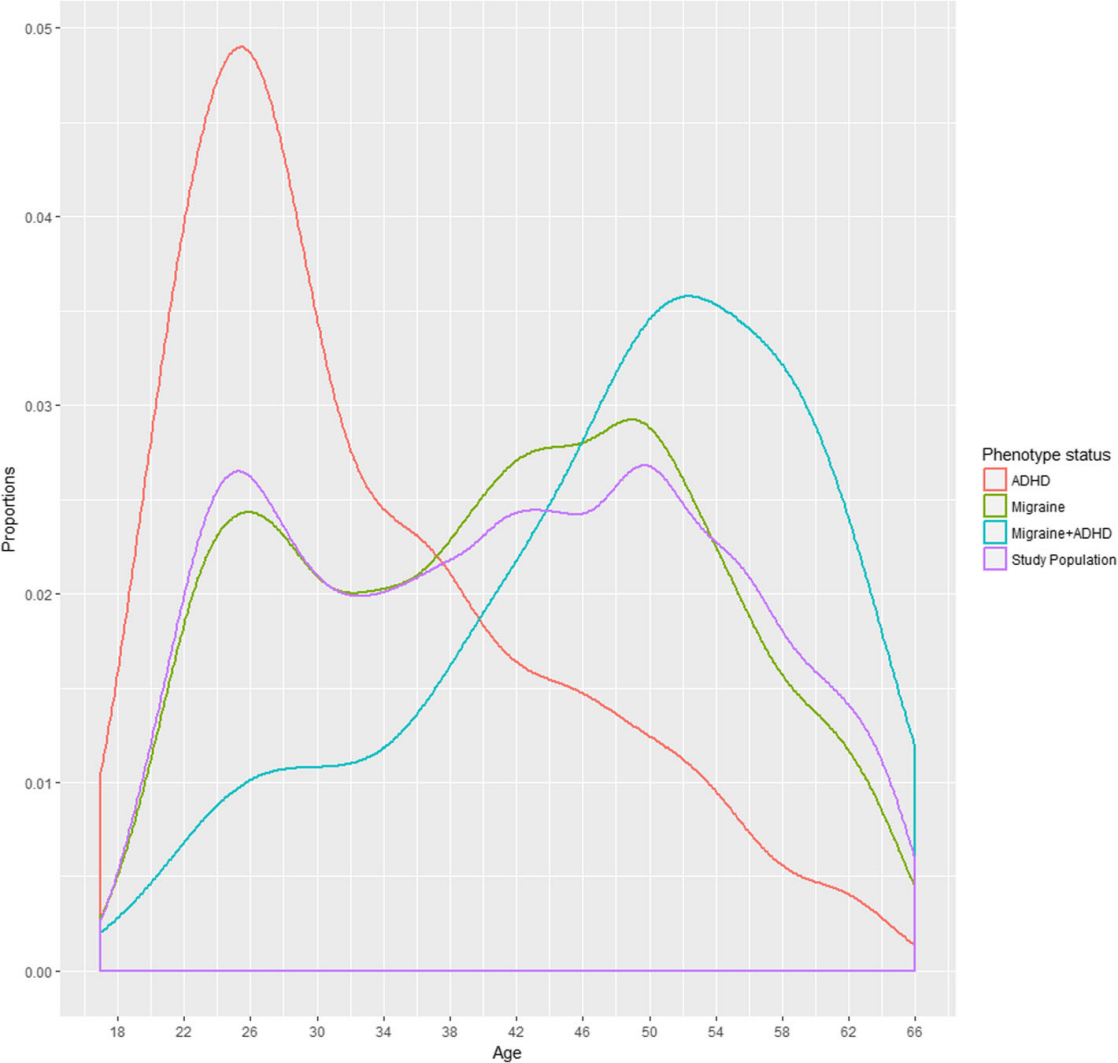
Age distribution. The X-axis shows age from 17 to 66 years, the Y-axis shows the proportional distribution by age of the study population, colored according to phenotype status on the right

The population consisted of 26,456 participants (median age: 42 years, IQR = 30–52 years) from the DBDS, of whom 24.2% screened positive for migraine (median age: 42 years, IQR = 31–51 years, female-to-male ratio: 1:0.6), 2.61% screened positive for ADHD (median age: 29 years, IQR = 25–40 years, female-to-male ratio: 1:1.4), and 0.90% reported having both migraine and ADHD (median age: 31 years, IQR = 24–41 years, male-to-female ratio: 1:0.74), see Table 1 for the prevalence. There was a significant difference in the age and sex distributions for participants with either migraine or ADHD (Wilcoxon test and chi-square exact, *P* < 1e-15 and *P* < 1e-15, respectively) (Fig. 1), thus we included a correction for age, sex and interaction of age and sex in the model.

**Table 1:** Characteristics of the study population (*N* = 26,456) with respect to migraine and ADHD symptoms

	N					
Description	Female (%^e^)		Male	Total	(%^f^)	*P*-value^d^
The total study population	12,247	(46.3)	14,209	26,456	(100)	
Migraine^a^	4024	(63.0)	2366	6390	(24.2)	< 0.001
- *Without visual disturbances*	2187	(62.0)	1343	3530	(13.3)	< 0.001
- *With visual disturbances*	1832	(64.2)	1019	2851	(10.8)	< 0.001
ADHD^b^	285	(41.3)	405	690	(2.6)	0.0085
- *Inattention*	83	(39.7)	126	209	(0.79)	0.06
- *Hyperactivity-impulsivity*	67	(43.0)	89	156	(0.59)	0.42
- *Screening items*^*c*^	182	(26.7)	400	682	(2.58)	< 0.001
Migraine and ADHD	137	(57.6)	101	238	(0.90)	< 0.001

^a^Symptoms of migraine was assessed by the SQM [19],^b^ ADHD symptoms were assessed by the ASRS [20], ^c^ Screening as described by Kessing et al. [23], ^d^ Test of gender differences of total population and subgroup by chi-square test, ^e^ percentage females of the subgrup, ^f^ percentage of the total sample

There was an association between migraine and ADHD symptoms with OR = 1.81 (95CI%: 1.53–2.12, *P* = 1.4e-14) including corrections for age, sex and the interaction of age and sex as covariates (Table 2a). As expected, male sex is protective (OR = 0.57), and increased age further increased protection with OR = 9.99 per year (Table 2a). To ease literature comparison, we present the sex-stratified results (Table 2b + c) and repeated the regression analysis using age bins (10 years), see Table 3. The association was not explained by one specific item in the ASRS questionnaire (data not shown). The highest risk was found for migraine with visual disturbances and ADHD symptoms (OR = 2.05, 95CI%: 1.55–2.68, *P* = 3.3e-11). Using the national prescription register we did not find any difference in frequency of triptan purchases with and without ADHD symptoms (at least one purchase: 22.1% vs 23.2%, *p* = 0.80, at least 2 purchases: 13.7% vs 14.4%, and at least 10 purchases: 3.2% vs 4.9%, *p* = 0.39).

**Table 2:** Multivariable Logistic regression analysis

*Model*		*a) All genders*			*b) Females only*			*c) Males only*		
Outcome	*Variable*	*OR*	*CI 95%*	*P-value*	*OR*	*CI 95%*	*P-value*	*OR*	*CI 95%*	*P-value*
Migraine	ADHD	1.81	1.53–2.12	< 0.001	2.01	1.58–2.54	< 0.001	1.64	1.30–2.06	< 0.001
	Age	1.00	1.00–1.01	0.001	1.01	1.00–1.01	< 0.001	1.00	0.99–1.00	0.051
	Gender (Male)	0.57	0.47–0.69	< 0.001						
	Age:Gender (Male)	0.99	0.99–0.99	< 0.001						
With Visual Disturbances	ADHD	1.98	1.61–2.41	< 0.001	2.05	1.55–2.68	< 0.001	1.89	1.38–2.53	< 0.001
	Age	1.00	1.00–1.00	0.84	1.00	1.00–1.00	0.11	1.00	0.99–1.00	0.11
	Gender (Male)	0.53	0.40–0.69	< 0.001						
	Age:Gender (Male)	1.00	0.99–1.00	0.16						
Without Visual Disturbances	ADHD	1.52	1.22–1.88	< 0.001	1.69	1.24–2.27	< 0.001	1.37	0.99–1.86	0.047
	Age	1.01	1.00–1.01	< 0.001	1.01	1.00–1.01	< 0.001	1.00	0.99–1.00	0.23
	Gender (Male)	0.63	0.49–0.82	< 0.001						
	Age:Gender (Male)	0.99	0.98–1.00	< 0.001						

Multivariable Logistic regression analysis of migraine and migraine with and without visual disturbances, subsequent stratified on gender.

Reference of the regression model is given in ()

**Table 3:** Multivariable logistic regression analysis

*Outcome*	*Variable*	*OR*	*CI 95%*	*P-value*
Migraine	ADHD	1,86	1,58-2,19	< 0.001
	Age 3X^a^	1,34	1,20-1,51	< 0.001
	Age 4X^a^	1,59	1,43-1,76	< 0.001
	Age 5X^a^	1,27	1,14-1,42	< 0.001
	Age 60 + ^a^	0,98	0,84-1,15	0.80
	Gender (Male)	0,49	0,44-0,56	< 0.001
	Gender (Male) interaction with:			
	Age 3X^a^	0,78	0,66-0,93	0.007
	Age 4X^a^	0,75	0,63-0,88	< 0.001
	Age 5X^a^	0,75	0,63-0,89	0.0013
	Age 60 + ^a^	0,84	0,66-1,07	0.16
With Visual Disturbances	ADHD	2,01	1,63-2,45	< 0.001
	Age 3X^a^	1,13	0,97-1,31	0.110
	Age 4X^a^	1,20	1,05-1,38	0.0095
	Age 5X^a^	1,13	0,98-1,31	0.090
	Age 60 + ^a^	0,78	0,63-0,97	0.028
	Gender (Male)	0,49	0,41-0,57	< 0.001
	Gender (Male) interaction with:			
	Age 3X^a^	0,90	0,71-1,15	0.41
	Age 4X^a^	0,83	0,66-1,05	0.13
	Age 5X^a^	0,79	0,62-1,01	0.06
	Age 60 + ^a^	1,12	0,80-1,57	0.5
Without Visual Disturbances	ADHD	1,59	1,28-1,97	< 0.001
	Age 3X^a^	1,47	1,27-1,70	< 0.001
	Age 4X^a^	1,81	1,59-2,07	< 0.001
	Age 5X^a^	1,34	1,16-1,54	< 0.001
	Age 60 + ^a^	1,15	0,94-1,39	0.16
	Gender (Male)	0,53	0,46-0,63	< 0.001
	Gender (Male) interaction with:			
	Age 3X^a^	0,73	0,58-0,92	0.0069
	Age 4X^a^	0,72	0,59-0,89	0.0026
	Age 5X^a^	0,75	0,60-0,93	0.011
	Age 60 + ^a^	0,69	0,50-0,94	0.021

^a^ Reference is age < 30

**Fig. 2:**
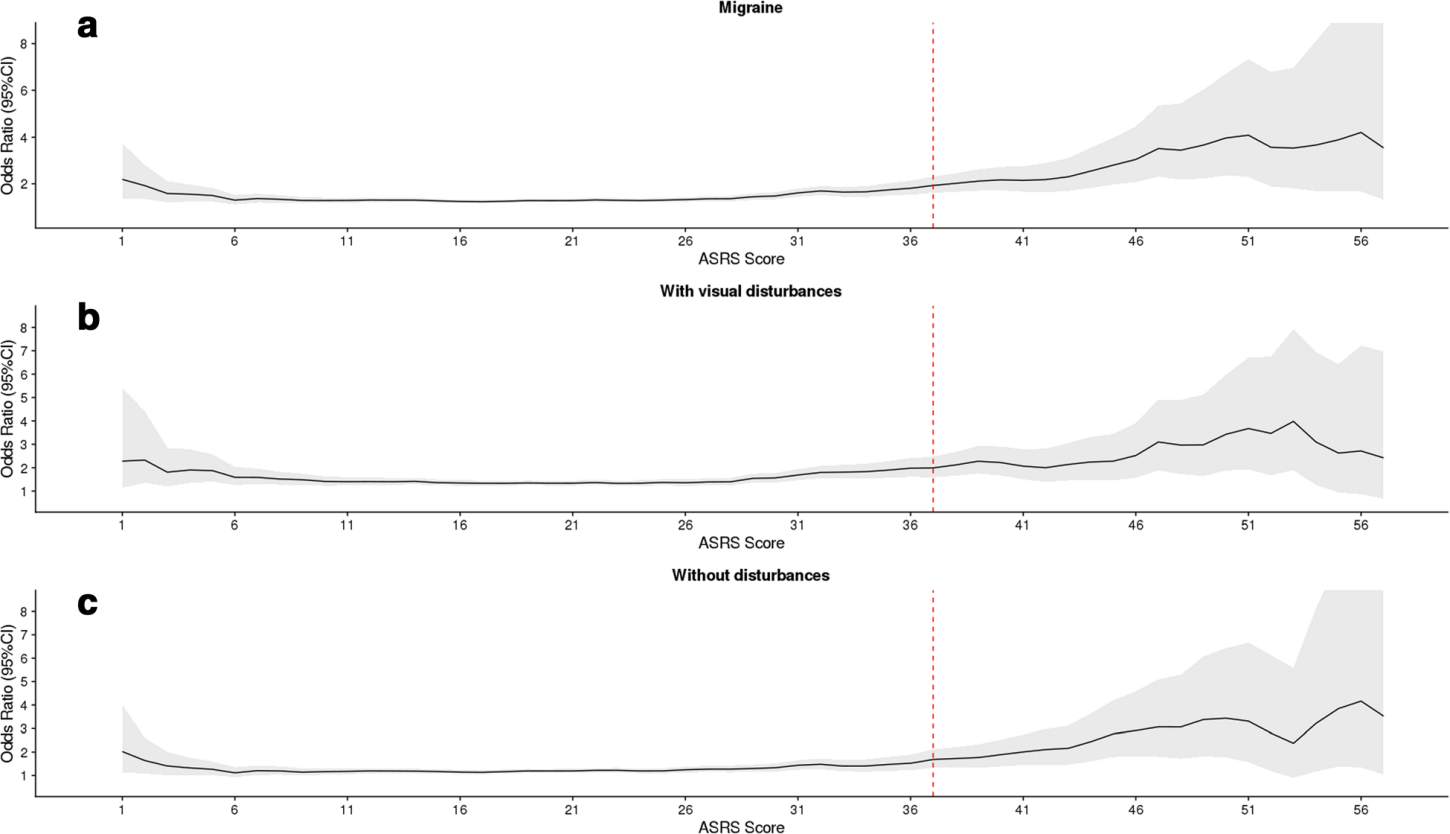
The odds ratio of the multivariable regression analysis at each ASRS score. X-axis presents the ASRS score. The vertical red dotted line marks the threshold for ADHD for the optimal 18-item score. Left Y-axis is the Odds Ratio indicated as the black line and the 95% confidence interval by the grey area. All analysis is done with multivariable logistic regression adjusting for age, sex and the interaction of age and sex

The association between migraine and ADHD symptoms was statistically significant irrespective of the employed ASRS score threshold (Fig. 2a), with a tendency to increase with the ASRS score. The same results were observed for ADHD symptoms and migraine with visual disturbances (Fig. 2b), however; no association was detected for migraine without visual disturbances with ASRS scores below 10 and above 51 (Fig. 2c).

**Fig. 3:**
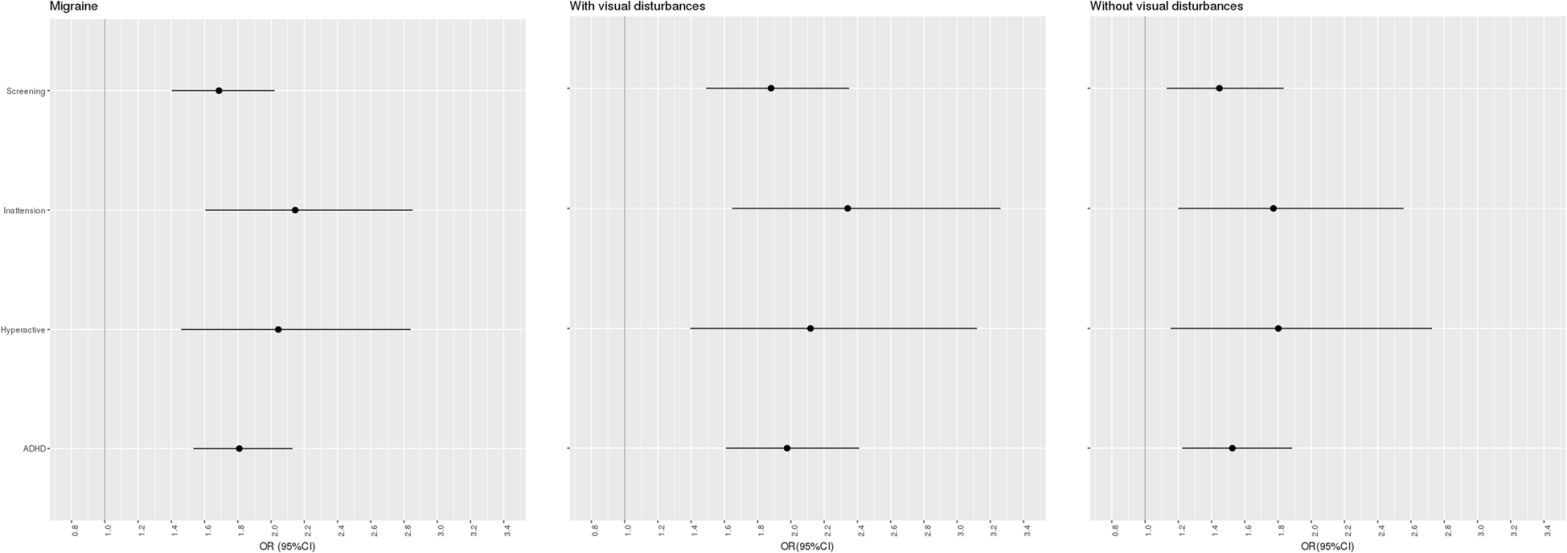
Analysis of ADHD and Migraine endophenotypes. X-axes are odds ratio (black dot) and 95% confidence intervals (black lines) with a grey vertical line indicating an odds ratio of 1. The three graphs display migraine, and migraine with and without visual disturbances. The Y-axis displays the ADHD and two ADHD endophenotypes

Migraine was associated with both ADHD endophenotypes (inattention and hyperactivity-impulsivity), with migraine with visual disturbances showing a marginally larger effect than migraine without visual disturbances, notably with overlapping confidence intervals (Fig. 3).

## Discussion

We address the comorbidity of migraine and ADHD symptoms in a healthy population of 29,489 adults using two clinically validated questionnaires (SQM and ASRS). Our results show a strong and statistically significant comorbidity between the two disorders which was irrespective of the threshold (i.e., not restricted to the commonly used ASRS score threshold of 37 (Fig. 2). The regression model clearly shows that being male protects against migraine and increased age is further protective as seen in the interaction between age and sex (Table 2). Stratified analyses suggested that the observed comorbidity was more pronounced in migraineurs experiencing visual disturbances; however, no differences in association with migraine were seen for the two ADHD endophenotypes (inattention and hyperactivity-impulsivity).

The current study significantly supplements the sparse literature regarding the comorbidity between migraine and ADHD in adults by using a large, healthy study population of individuals who have not been exposed to chronic treatment for migraine or for ADHD. It supports the meta-analysis by Salem et al., showing OR of 1.3, consisting primarily of child and adolescence studies. This meta-analysis cohort consisted of a clinical case-control cohort, (*n* = 572) with ADHD [16], and a cross-sectional study using prescription data on anti-migraine and anti-ADHD drugs from the entire Norwegian population [17]. Thus, the comorbidity between migraine and ADHD seems present both in and out of a clinical setting. The origin of the comorbidity is not obvious and typical prognostic features such as the age of onset, and the female-to-male ratio of the two diseases are somewhat contradicting. It has previously been suggested that the co-occurrence of migraine and ADHD originates from common pathophysiological mechanisms potentially related to dysfunctions in the dopaminergic system [16, 17, 31]. This arises because many of the migraine symptoms, including prodromal symptoms, can be provoked with dopamine receptor stimulation, and some can even be quantified in rat models with dopaminergic activiation [31]. Dopamine has long been thought to be involved in ADHD pathology, and the effective ADHD drug, methylphenidate, acts by inhibiting dopamine reuptake. However, the comorbidity could also be more complex because of common co-morbidities of other psychiatric disorders such as anxiety and mood disorders [13, 32–36]. Their etiological comorbidity may arise because of pleiotropic factors. This has recently been supported by Antilla et al., showing a significant genetic correlation between migraine and ADHD [37]. Interestingly, major depressive disorder was also found to significantly correlate with both migraine and ADHD suggesting that common pleiotropic factors exist.

The observed prevalence of migraine and ADHD (34.7%) was higher than reported by Fasmer et al. (28.3%) in a sample of ADHD patients with similar age and sex distribution [16]. However, there is an anecdote among headache individuals with headache that donating blood gives symptom relief, which could explain the frequency of migraine, i.e. bloodletting by phlebotomy. Furthermore, we used self-reported ADHD rather than formal clinical diagnoses in our analysis, which may influence the results with false positive and negative cases. Despite these methodological differences, we found the prevalence of self-reported migraine and ADHD symptoms in males (24% vs. 23%) to be like that reported by Fasmer et al. [16].

We found that the comorbidity between migraine and ADHD was most prevalent among participants peaking at 52 to 53 years of age, and in the 40 decade compared to the 17–29 age group, which is somewhat inversely correlated with the age distribution of ADHD symptoms in the study population (Fig. 1). This could imply that the manifestations of comorbid migraine and ADHD occur rather late in life when compared to ADHD in general, or that ADHD symptoms mask the presence of migraine in the younger participants. Similar results have been reported by Fasmer et al. [17] using simultaneous prescriptions of anti-migraine and anti-ADHD medication as a proxy for comorbidity. However, this was not true for the 10–19 years age group, suggesting that ADHD among adolescents may have no comorbidity with migraine and thus having a distinct etiopathology or, as previously mentioned, there is a masking of migraine symptoms in young individuals with ADHD. The notion of a different etiopathology among ADHD patients is suggested by Johansson et al. (Johansson et al. 2008), showing that susceptibility genes differ between persistent and juvenile ADHD [38]. Furthermore, the transition of ADHD symptoms from childhood to adulthood is well recognized where the hyperactivity-impulsivity often becomes less prominent.

We did not detect a specific association between any ADHD endophenotypes and migraine which is congruent with previous reports for children, e.g., Pakainis et al. and Arruda et al [19, 39] It is noteworthy that we do not see a large difference between ADHD inattention and hyperactive endophenotype. Although speculative, the smaller difference between hyperactive and inattention endophenotype in the study cohort could reflect that the inattention is more often referred to treatment and thus are not permitted to donate blood [40].

Participants reporting migraine preceded by visual disturbances (migraine with visual aura symptoms) were more often affected by co-occurring ADHD symptoms when compared to participants reporting no visual disturbances and combined (Table 2, Fig. 2). No obvious shared mechanism between visual disturbances and ADHD symptoms is known. The neurovascular phenomenon cortical depression spreading is suggested as the pathophysiology of aura symptoms in migraine patients [41], however, there are no published studies on ADHD patients. Ophthalmologic disturbances appear to be more frequent in patients diagnosed with ADHD [42], however, it is very speculative whether this could explain the observed comorbidity and further studies are needed.

The strength of the study is the large sample size and that chronic treatment of migraine and ADHD do not influence the results, e.g., treatment-induced migraine attacks. Furthermore, we used validated questionnaires to assess migraine and ADHD symptoms. In accordance with Danish clinical guides we use the recommended version of ASRS to assess ADHD. While a new version based on DSM V exists, a recent study found almost identical specificity and sensitivity of the two versions [43]. Furthermore, we show that 89% of individuals using triptans are captured, and the prevalence of triptan users in migraineurs with and without ADHD symptoms is the same. When interpreting the results, it is important to take into consideration that the study population is a healthy donor population, presumably producing a conservative estimate of the association as individuals exposed to chronic medication or chronic illness are excluded as donors. Further, we suggest carrying out longitudinal cohort and twin studies to assess causality.

## Conclusion

We demonstrate a significant corbobidity between migraine and ADHD in adults, and this is most prominent for participants with migraine with visual disturbances. These results contribute to the understanding of genetic correlation seen between ADHD and migraine and seeds future studies that will elucidate which genetic and environmental factors contribute to migraine-ADHD comorbidity.

## Article highlights

- This study examined the association between migraine and ADHD in a large adult population
- There is a strong association between migraine and ADHD (odds ratio = 1.8, 95% CI = 1.5–2.1)
- The association between migraine and ADHD seems strongest for individuals with visual disturbances

## Abbreviations

95%CI95: Confidence interval
ADHD: Attention Deficit and Hyperactivity Disorder
ASRS: ADHD Self-Report Scale
IQR: Interquartile range
OR: Odss Ratio
SQM: Screening questionnaire for migraine
WHO: World Health Organization
